# Characterizing the virome of *Ixodes ricinus* ticks from northern Europe

**DOI:** 10.1038/s41598-017-11439-y

**Published:** 2017-09-07

**Authors:** John H.-O. Pettersson, Mang Shi, Jon Bohlin, Vegard Eldholm, Ola B. Brynildsrud, Katrine Mørk Paulsen, Åshild Andreassen, Edward C. Holmes

**Affiliations:** 1 Marie Bashir Institute for Infectious Diseases and Biosecurity, Charles Perkins Centre, School of Life and Environmental Sciences and Sydney Medical School, the University of Sydney, Sydney, New South Wales 2006 Australia; 2 Infectious Disease Control and Environmental Health, Norwegian Institute of Public Health, Oslo, Norway; 3 Department of Microbiology, National Veterinary Institute, Uppsala, Sweden; 4 Department of Medical Biochemistry and Microbiology (IMBIM), Zoonosis Science Center, Uppsala University, Uppsala, Sweden; 5 Norwegian University of Life Sciences, Faculty of Veterinary Medicine, Department of Production Animal Clinical Sciences, Oslo, Norway

## Abstract

RNA viruses are abundant infectious agents and present in all domains of life. Arthropods, including ticks, are well known as vectors of many viruses of concern for human and animal health. Despite their obvious importance, the extent and structure of viral diversity in ticks is still poorly understood, particularly in Europe. Using a bulk RNA-sequencing approach that captures the complete transcriptome, we analysed the virome of the most common tick in Europe – *Ixodes ricinus*. In total, RNA sequencing was performed on six libraries consisting of 33 *I. ricinus* nymphs and adults sampled in Norway. Despite the small number of animals surveyed, our virus identification pipeline revealed nine diverse and novel viral species, phylogenetically positioned within four different viral groups – bunyaviruses, luteoviruses, mononegavirales and partitiviruses – and sometimes characterized by extensive genetic diversity including a potentially novel genus of bunyaviruses. This work sheds new light on the virus diversity in *I. ricinus*, expands our knowledge of potential host/vector-associations and tick-transmitted viruses within several viral groups, and pushes the latitudinal limit where it is likely to find tick-associated viruses. Notably, our phylogenetic analysis revealed the presence of tick-specific virus clades that span multiple continents, highlighting the role of ticks as important virus reservoirs.

## Introduction

Viruses are present in all domains of life^1, 2^. Arthropods are a particularly rich source of viruses, with multiple novel viruses having recently been identified through bulk RNA-sequencing approaches (so-called ‘meta-transcriptomics’)^1, 3^. One group of arthropods that are especially relevant for human and veterinary health are ticks (Order: Ixodida). Three families of ticks are recognised – Argasidae (argasids, soft ticks), Ixodidae (ixodids, hard ticks), and Nuttalliellidae – that together comprise close to 900 species, of which approximately 700 recognised species are hard ticks^4^.

**Figure 1:**
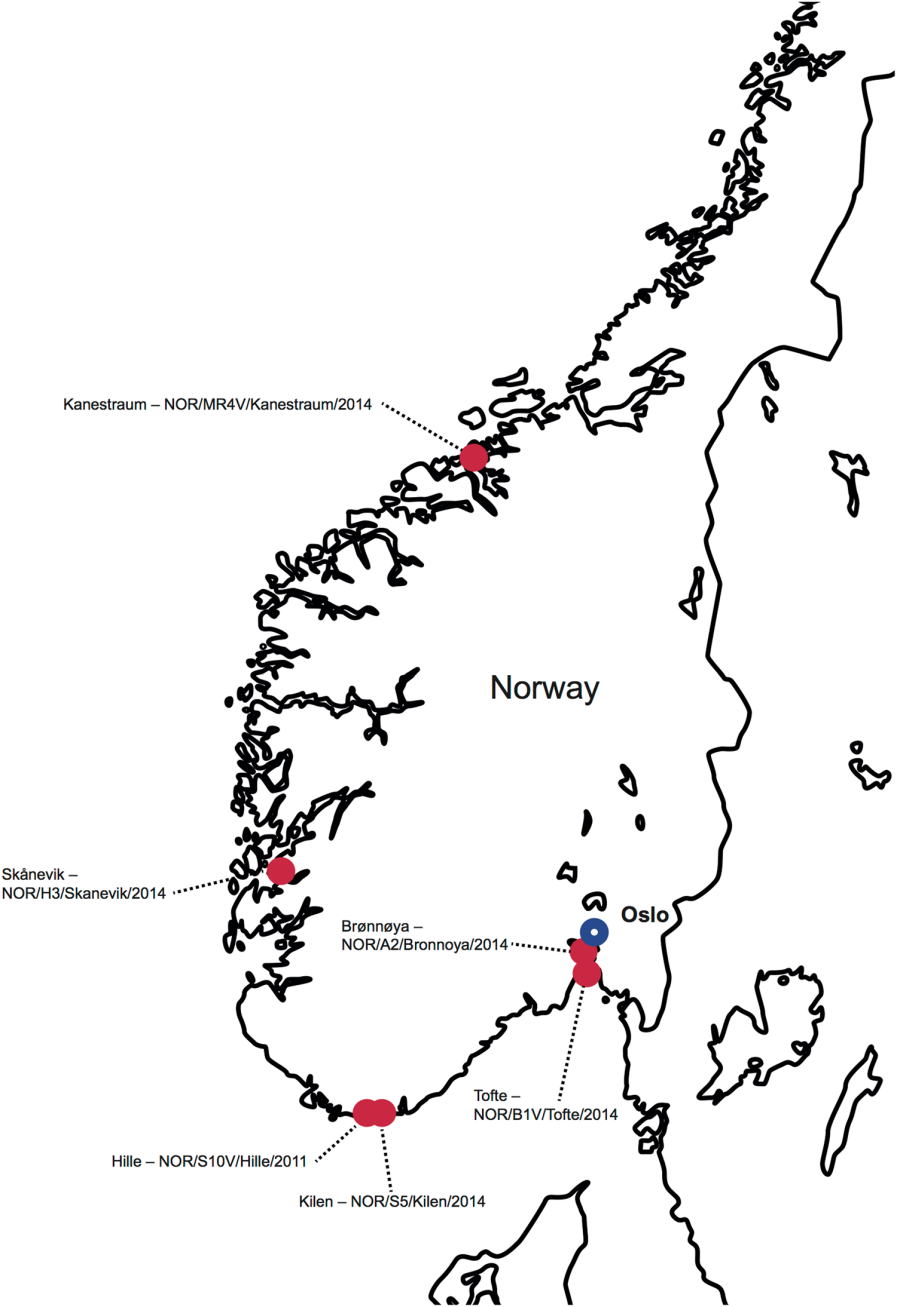
Map of Norway showing the collection sites for all tick samples. The map is a modified version of the original (http://english.freemap.jp/item/europe/narway.html), licensed under a Creative Commons Attribution 3.0 Unported License (https://creativecommons.org/licenses/by/3.0/).

Although ixodid ticks are well known to be important vectors of human and animal bacterial and viral pathogens, such as *Rickettsia* spp. and Crimean-Congo Haemorrhagic Fever virus^5, 6^, little is known about the viral diversity carried by ixodid ticks on a global scale. Virome studies of ticks collected in Asia and North America have revealed a high diversity of RNA viruses. The novel viruses represent most recognised viral families and are likely to include previously uncategorised pathogenic human and veterinary viruses, as well as viruses that may be considered commensal^1, 3, 7, 8^. In Europe, Ixodid ticks, and *Ixodes ricinus* in particular, are known to be vectors of tick-borne encephalitis virus and Louping-ill virus^9–11^ that cause important diseases in mammalian populations. However, far less is known about the biodiversity of viruses present in ixodid ticks in the absence of an association with a specific vertebrate disease. To address this question we performed high-throughput RNA sequencing on *I. ricinus* ticks collected predominantly in southern Norway (Fig. 1). From these data were we able to recover complete meta-transcriptomes and determine the viral diversity present in these ticks.

## Results/Discussion

**Figure 2:**
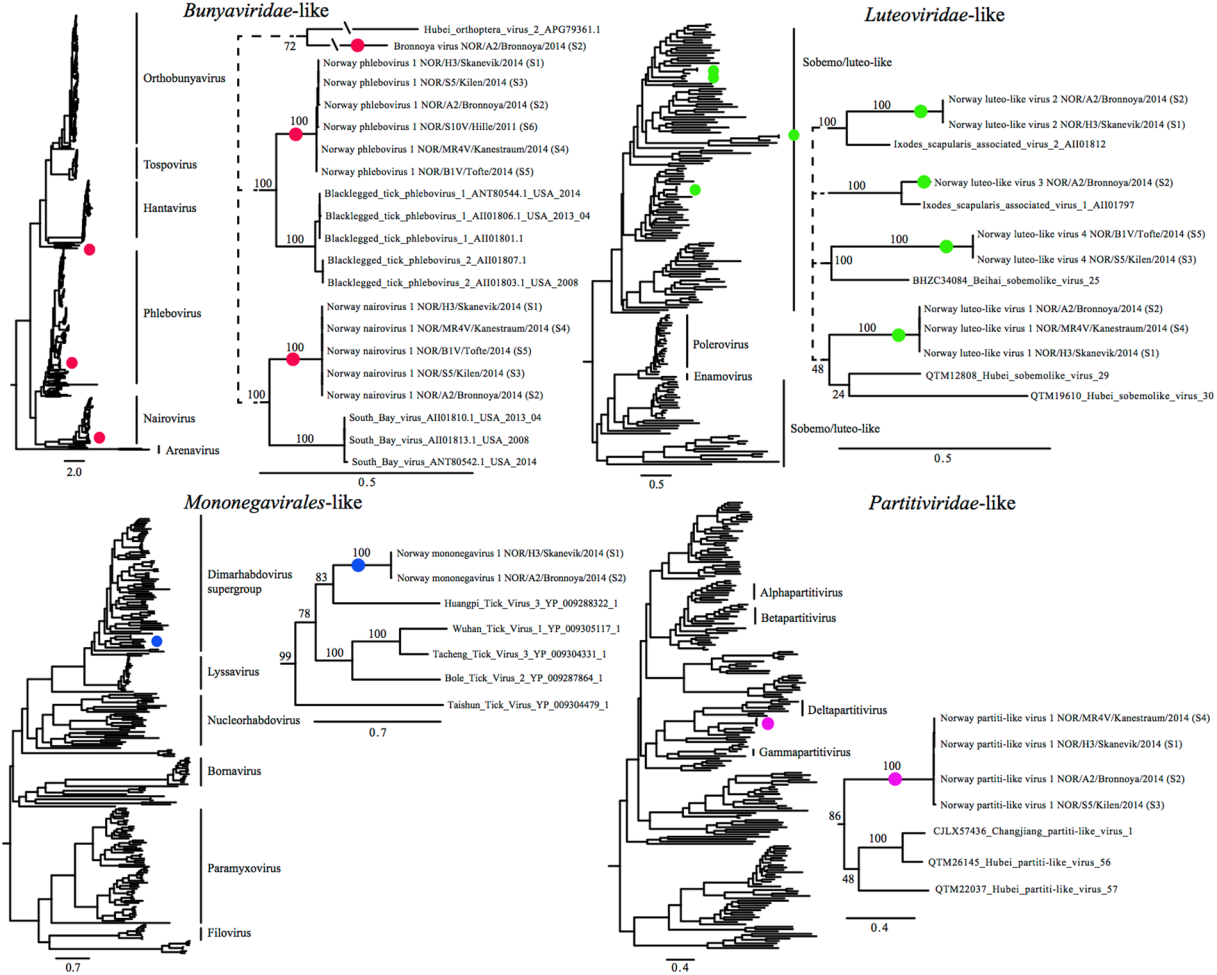
Family-wide phylogenetic trees of the RdRp segments based on representative amino acid sequences from four viral families, the *Bunyaviridae*, *Luteoviridae*, *Mononegavirales* and *Partitiviridae*, and sequences produced in this study (as labelled). Values on branches indicate bootstrap support based on 1000 bootstrap replicates. All branches are scaled according to the number of amino acid substitutions per site. The trees were mid-point rooted for purposes of clarity only.

We used a meta-transcriptomics approach to characterise the virome of *I. ricinus* ticks collected in Norway (a map of sampling sites is shown in Fig. 1). Accordingly, close to 1500M bp were generated for six *I. ricinus* RNA sequencing libraries, including 30 *I. ricinus* nymphs and 3 *I. ricinus* adults, pooled by geographical location. After quality trimming these libraries produced after *de novo* assembly a total of 735,413 contigs, from which we identified 26 unique virus RNA-dependent RNA polymerase (RdRp)-sequences and, for a subset, associated structural genes (Suppl. Fig. S1, Suppl. Table 1). Overall, these data comprised nine novel and divergent tick-associated RNA viruses, taxonomically positioned within or close to members of the *Bunyaviridae*, *Luteoviridae*, *Mononegavirales* and *Partitiviridae* (Fig. 2). Each of the six libraries contained between 1–7 viruses (Table 1, Suppl. Table 1), with amino acid identity ranging from between 28–90% compared to the most similar viral sequences published previously (Table 1), indicating that these are indeed novel viruses. Furthermore, the majority of the viruses detected were abundant in all libraries, with relative frequencies of viral contigs (in percentage; the number of reads assembled to each virus contig in relation to the total number of reads per library) ranging from <0.01–2.62%, and with mean sequencing depths (as calculated by the number of reads per nucleotide position in relation to the total length of the contig) ranging from ×7−x24,683 (Table 1, Suppl. Table 1). In some cases all the genes of a specific virus could not be clearly identified, likely because high levels of sequence divergence can make it difficult to identify genes other than the relatively well conserved RdRp with a high degree of certainty. It is also possible that the virus genes in question were at low abundance in the libraries and/or only partial in structure which also compromised identification.

**Table 1:** Comparison of amino acid identity, contig length and mean coverage and relative frequency between the viruses identified in this study. Library S1–S3 corresponds to nymphal pools NOR/H3/Skanevik/2014, NOR/A2/Bronnoya/2014 and NOR/S5/Kilen/2014, respectively, and libraries S4–S6 corresponds to individual adults NOR/MR4V/Kanestraum/2014, NOR/B1V/Tofte/2014 and NOR/S10V/Hille/2011, respectively.

**Virus family**	**Virus**	**# of unique contigs**	**Contig length (nts)**	**Present in libraries**	**RdRp mean seq. depth**	**RdRp relative frequency (%)**	**RdRp % idenity**	**Nearest hit (accession number)**
*Bunyaviridae*	Bronnoya virus	1	9185	S2	51	0.01	28	Hubei orthoptera virus 2 (APG79361.1)
*Bunyaviridae*	Norway nairovirus 1	5	12239–15089	S1–S5	650–1645	0.25–0.47	65	South Bay virus (AII01810.1)
*Bunyaviridae*	Norway phlebovirus 1	6	6711–6746	S1–S6	105–4732	0.02–0.45	75	Blacklegged tick phlebovirus 1 (AII01801.1)
*Luteoviridae*	Norway luteo-like virus 1	3	3250–3252	S1, S2, S4	23–24683	<0.01–2.62	63	Hubei sobemo-like virus 29 (YP009330084.1)
*Luteoviridae*	Norway luteo-like virus 2	2	2632–2647	S1, S2	5–451	<0.01–0.07	78	Ixodes scapularis associated virus 2 (AII01812.1)
*Luteoviridae*	Norway luteo-like virus 3	1	2005	S2	63	<0.01	90	Ixodes scapularis associated virus 1 (AII01797.1)
*Luteoviridae*	Norway luteo-like virus 4	2	3440–5972	S3, S5	10–45	<0.01	57	Beihai sobemo-like virus 25 (YP009336811.1)
*Mononegavirales*	Norway mononegavirus 1	2	11506–11694	S1, S2	33–69	0.01–0.02	47	Huangpi Tick Virus 3 (YP009288322.1)
*Partitiviridae*	Norway partiti-like virus 1	4	1026–1726	S1–S4	7–94	<0.01	52	Hubei partiti-like virus 56 (APG78242.1)

The most divergent new virus was found within the *Bunyaviridae*. Bunyaviruses are segmented negative-stranded viruses that include at least five different genera – Hantavirus, Nairovirus, Negevirus, Orthobunyavirus, and Phlebovirus – many of which include human pathogens^12^. Recently, novel bunyaviruses have been discovered in a variety of organisms, including ticks, some of which are pathogenic^13–15^, such as the Heartland virus and the severe fever with thrombocytopenia syndrome virus^16^. In addition, it was previously demonstrated that phlebo- and nairoviruses are present in *I. ricinus* ticks sampled in France^17^. We identified three novel viruses within the *Bunyaviridae*, denoted here as Bronnoya virus, Norway nairovirus 1 and Norway phlebovirus 1 (Fig. 2, Suppl. Figure 2). Bronnoya virus was the most divergent virus found in our study, exhibiting only 28% amino acid identity to the most similar published virus sequence – Hubei orthoptera virus 2 (APG79361.1) – and tentatively positioned between the Phleboviruses and Hantaviruses (Fig. 2, Suppl. Figure 2). Due to its high divergence compared to other bunyaviruses, we suspect that it likely represents a novel genus within *Bunyaviridae*, although whether it is truly tick-associated remains to be determined. The other two viruses, Norway nairovirus 1 and Norway phlebovirus 1, fell within the genera Nairovirus^18^ and Phlebovirus^19^, respectively. In particular, Norway nairovirus 1 was most closely related to South Bay virus while the closest relative of Norway phlebovirus 1 was Blacklegged tick phlebovirus 1, both of which were described in *Ixodes scapularis* ticks collected in North America^7^. These two viruses were also the most common viruses found in the data set analysed here: Norway nairovirus 1 was present in five of the six libraries and Norway phlebovirus 1 was present in all six libraries, both of which were abundant within the tick libraries, with relative frequencies of viral contigs ranging between 0.02–0.47%, and with mean sequence depths ranging from to ×105−x8,492 (Table 1., Suppl. Table 1). Hence, the combination of these abundance data and phylogenetic history of tick associations suggests that these viruses are tick-associated and likely present in diverse geographic regions.

Luteoviruses are a family of single-stranded positive-sense RNA viruses commonly known to include many economically important plant pathogens^20, 21^. More recently, Luteo-like viruses, (i.e. related, but phylogenetically divergent compared to the currently recognised members of *Luteoviridae*), have been discovered in diverse invertebrates including clams, dragonflies, octopuses, ticks and spiders^1^, indicating that this group of viruses has a wider array of host-associations than plants alone. In support of this notion we identified four novel luteo-like viruses, denoted Norway luteo-like virus 1–4, in our tick samples. Of these, Norway luteo-like virus 2–3, cluster with *Ixodes scapularis* associated viruses, while Norway luteo-like virus 1 and 4, were most closely related to dragonfly- and clam-associated viruses from China, respectively (Fig. 2, Suppl. Fig. 3). The abundance estimates for Norway luteo-like virus 1 (relative frequency: 2.62%; mean sequence depth: ×24,683) suggest that this is most likely a tick-associated virus (Table 1, Suppl. Table 1). Norway luteo-virus 2–3 may also be tick-associated viruses as they group with *I. scapularis*-associated viruses in the phylogeny, with Norway luteo-virus 2 also being relatively abundant in one library (relative frequency: 0.07; mean sequence depth: ×878). For Norway luteo-virus 4, the association with ticks as hosts is less certain, in part because it was most closely related to clam-associated luteo-like viruses and because it was present at lower abundance (relative frequency: <0.01%; mean sequence depth: ×10 and ×45 in library three and five, respectively; Suppl. Table 1).

In two tick samples we identified a virus, denoted Norway mononegavirus 1, that fell in a clade comprising tick-associated viruses from China, positioned within the dimarhabdovirus supergroup^22^ of the family *Rhabdoviridae* (order *Mononegavirales* ^23^) (Fig. 2, Suppl. Fig. 4). The rhabdoviruses are a diverse set of single-stranded negative sense RNA viruses, known to infect both animals and plants^24^, with frequent host-switching during their evolution history^25^. Although there is relatively high sequence divergence between the viruses in the clade containing Norway mononegavirus 1, implying that much of the diversity is yet unsampled, all the viruses sampled in this clade have been isolated from ticks. However, compared to the nairo- and phleboviruses, the Norway mononegavirus 1 is less abundant within the libraries (Suppl. Table 1), so that any possible association with ticks need to be interpreted carefully.

Partitiviruses are double-stranded RNA viruses mostly known to be associated with plants and fungi, some of which are pathogens^26, 27^. Recently, meta-transcriptomic studies have revealed more diversity in this group of viruses, greatly expanding their known host range. Notably, several lineages of the partitiviruses appear to be abundant in arthropods, and the genomes of these hosts also harbour related endogenous virus elements in host genomes; hence, both these observations suggest that partitiviruses are commonly associated with arthropods^1^. We identified a highly divergent partiti-like virus, Norway partiti-like virus 1, in four of our samples (Fig. 2, Suppl. Fig. 5, Suppl. Table 1). Interestingly, this virus formed a cluster with dragonfly- and crayfish-associated viruses, which shared a close relationship with the endogenous viral elements found in plant lice (*Pachypsylla venusta*). Whether Norway partiti-like virus 1 is a truly tick-associated virus will need to be examined further.

The recent development of high-throughput RNA sequencing and meta-transcriptomics provides the opportunity to capture and quantify the complete virome of an organism or environmental sample, revolutionizing studies of virus ecology and evolution^1, 3^. A hallmark of RNA sequencing is its ability to provide a unbiased snap-shot of all viruses, and other RNA transcripts, that are actively expressed in a sample^28^. Utilising this technique we have demonstrated the presence of highly divergent novel RNA viruses in *I. ricinus* ticks collected from Norway. Although none of the viruses identified here can currently be definitively associated with any host/vector-species, the fact that the library abundance and mean sequence read depth of in particular Norway nairovirus 1, Norway phlebovirus 1 and Norway luteo-like virus 1 was high, suggests that these are very likely tick-associated viruses. In addition, as Norway nairovirus 1 and Norway phlebovirus 1 were also present in most sequence libraries, with samples originating from relatively geographically distant localities in Norway, it is also possible that these viruses are relatively common in *I. ricinus* ticks. Clearly, however, future studies need to be undertaken to determine if these viruses are present and prevalent in other geographic regions. Also of note was that these viruses were found between latitude 57.98 to 63.06 north. To our knowledge this study, particularly the locality of Kanestraum (at latitude 63.06), is the most northerly locality where phlebo-, nairo-, partiti-like-, and luteo-viruses have been detected.

It is evident that the currently known viral biodiversity has been systematically under-sampled and underestimated. Clearly, meta-transcriptome sequencing enables more intricate studies aimed at understanding the fundamental patterns and processes of virus ecology and evolution. Although the sample size of ticks used in this study was relatively small, it was striking that so many novel viruses were identified from a geographically restricted sampling location, mainly southern Norway, matching the diversity of RNA viruses previously found in Asia and North America. Clearly, such a relative abundance of viruses suggests that we are still only scratching the surface of the biodiversity of those viruses present in ticks on a global scale, and that larger and more systematic studies of *I. ricinus* are needed to reveal viruses that may be pathogenic to vertebrates.

## Methods

### Sample collection

Questing *Ixodes ricinus* nymphs and adults were collected by flagging the vegetation in different localities of Norway (Fig. 1) and stored in −80 °C until further processing. Following species identification, total nucleic acid/RNA was extracted as previously described^29^ from three nymph pools containing 10 nymphs each, and separately from the three individual adult ticks. Crucially, a cold-chain was maintained during all laboratory work.

### Library preparation and sequencing

For all libraries, ribosomal RNA (rRNA) was removed using the Ribo-Zero Gold (epidemiology) Kit (Illumina) following the manufacturer’s instructions. Subsequently, libraries were constructed for all rRNA-depleted RNA-samples using the KAPA Stranded RNA-Seq Kit (KAPA biosystems / Roche) with barcode adapters from Bioo Scientific, following the manufacturer’s instructions. Library cDNA-levels were quantified before, during and after library preparation with Qubit (ThermoFisher Scientific) high sensitive RNA/DNA assays and the fragment sizes were checked with a Bioanalzyer (Agilent Technologies). Sequencing libraries were subsequently pooled in equimolar amounts. All libraries were sequenced on a single lane (paired-end, 125 bp read-length) on an Illumina HiSeq. 2500 platform at the Norwegian Sequencing Centre (www.sequencing.uio.no).

### Quality checking, trimming and de novo assembly

Each RNA sequence library was quality trimmed with trim galore (www.bioinformatics.babraham.ac.uk/projects/trim_galore/) and then assembled *de novo* using Trinity v.2.2.0^30^.

### Virus discovery and genome annotation

Trinity assemblies were screened with blastX against a local database including all available (as of October 2016) protein sequences of reference RNA viruses as well as those recently published^1^, with hits with an *e*-value of 1 × 10^−5^ or better collated. As an additional screening test, all potential virus assemblies were screened against the Conserved Doman Database (www.ncbi.nlm.nih.gov/Structure/cdd/wrpsb.cgi) with an expected value threshold of 1 × 10^−3^ to identify viral gene segments. To exclude possible endogenous viruses, all virus assemblies were blasted against the *I. ricinus* reference genome (GCA_000973045.2). Finally, to assess mean sequence depth and relative frequency, the quality trimmed libraries were mapped back against all viral assemblies and the COX1^1^ mitochondrial gene of *I. ricinus* (KF197136.1) using Bowtie2 v.2.2.8^31^. Following previous studies^1^ we assume that a higher relative frequency suggests that the virus is more likely to be associated with ticks (rather than being a component of diet or environment). In the case of potentially multiple and/or overlapping open reading frames, FSFinder2 (http://wilab.inha.ac.kr/fsfinder2/) was used to identify possible ribosomal frameshifts.

### Multiple sequence alignments and evolutionary analysis

To infer the evolutionary relationships of the viruses discovered in this study, the protein translated RdRp open reading frame segments produced in this study were combined with representative complete proteomes and/or RdRp-segments of the *Bunyaviridae*, *Luteoviridae*, *Mononegavirales* and *Partitiviridae* were retrieved from NCBI Genbank (www.ncbi.nlm.nih.gov/genbank) and aligned using Mafft v.7.266^32^, employing the E-INS-i algorithm. Ambiguous regions in the alignments were removed with TrimAl v.1.2^33^. Following sequence alignment, ProtTest v.3.4^34^ was employed to select the best-fit model of amino acid substitution. Finally, maximum likelihood trees for all four alignments were inferred using the best-fit model of amino acid substitution (LG + I + Γ + F for all four alignments) with 1000 bootstrap replicates employing the PhyML v.3 program^35^. Phylogenetic trees were edited and visualised with FigTree v.1.4.2 (http://tree.bio.ed.ac.uk/software/figtree). All phylogenetic trees were mid-point rooted for purposes of clarity only.

### Data availability

All sequence reads generated in this project are available under the NCBI Short Read Archive (SRA) under accessions SRR5667127–SRR5667132 (BioProject ID: PRJNA390076) and all consensus virus genome sequences have been deposited in GenBank (accession numbers: MF141040–MF141077). Trimmed multiple sequence alignments of bunya-, luteo-, mononega- and partiti-like viruses are available as supplementary data (Supp. Data 1–4, respectively).

## Electronic supplementary material

Supplementary figure 1 to 5, Supplementary table 1

Dataset 1 to 4, Bunya, Luteo, Mononega, Picorna
