# Supplementary material for: Characterizing the virome of Ixodes ricinus ticks from northern Europe
Source: Sci Rep. 2017 Sep 7;7:10870. doi: 10.1038/s41598-017-11439-y (PMC5589870; doi:10.1038/s41598-017-11439-y)
Supplement: Supplementary file 1 — Supplementary figure 1 to 5, Supplementary table 1 [file 41598_2017_11439_MOESM1_ESM.pdf]

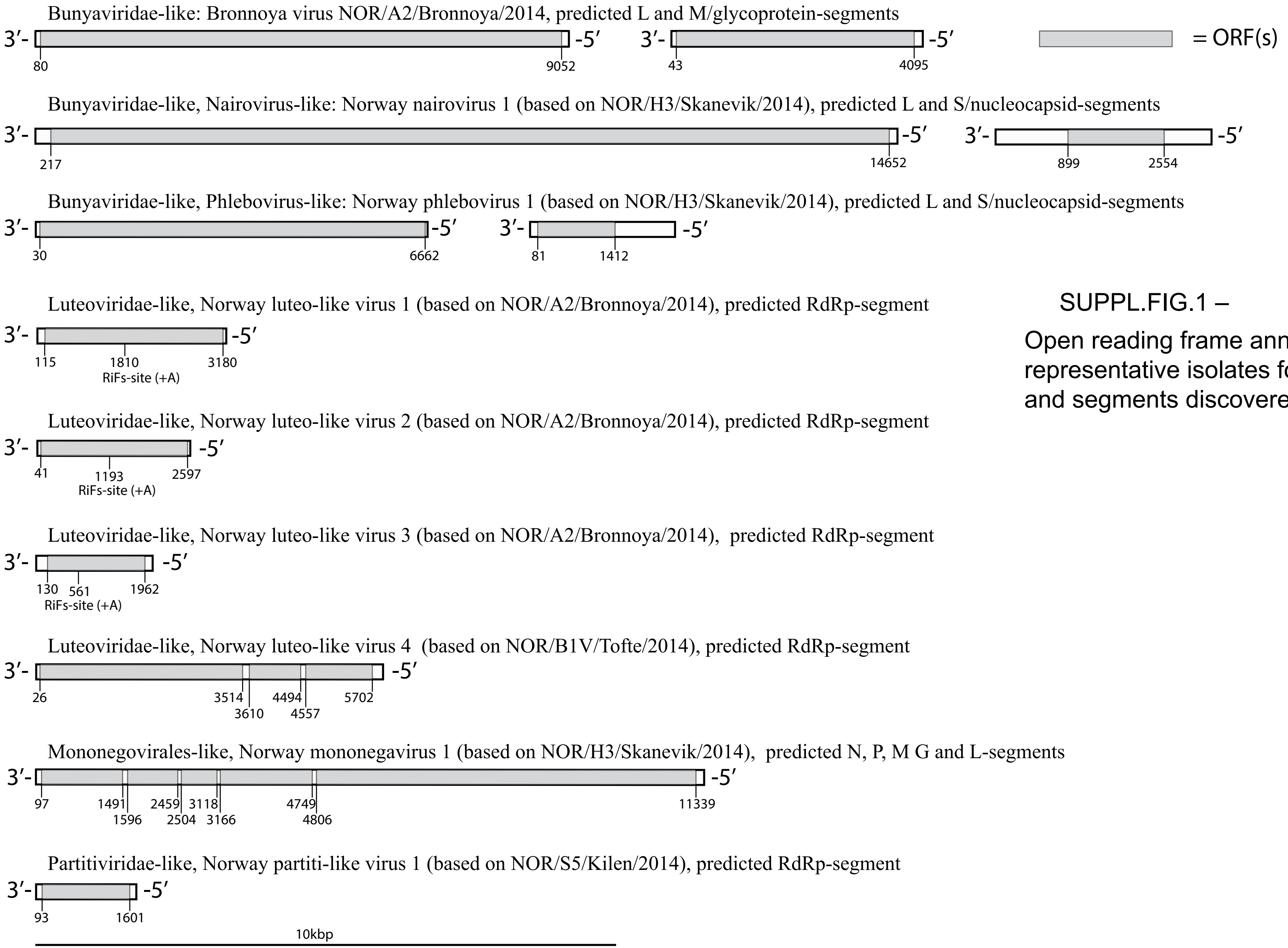

SUPPL.FIG.1 –

Open reading frame annotation of representative isolates for all viruses and segments discovered.

SUPP.FIG.2 – Complete maximum likelihood phylogenetic tree of all bunyavirus sequences. All branches are scaled according to the number of amino acid substitutions per site and the tree was mid-point rooted for clarity only.

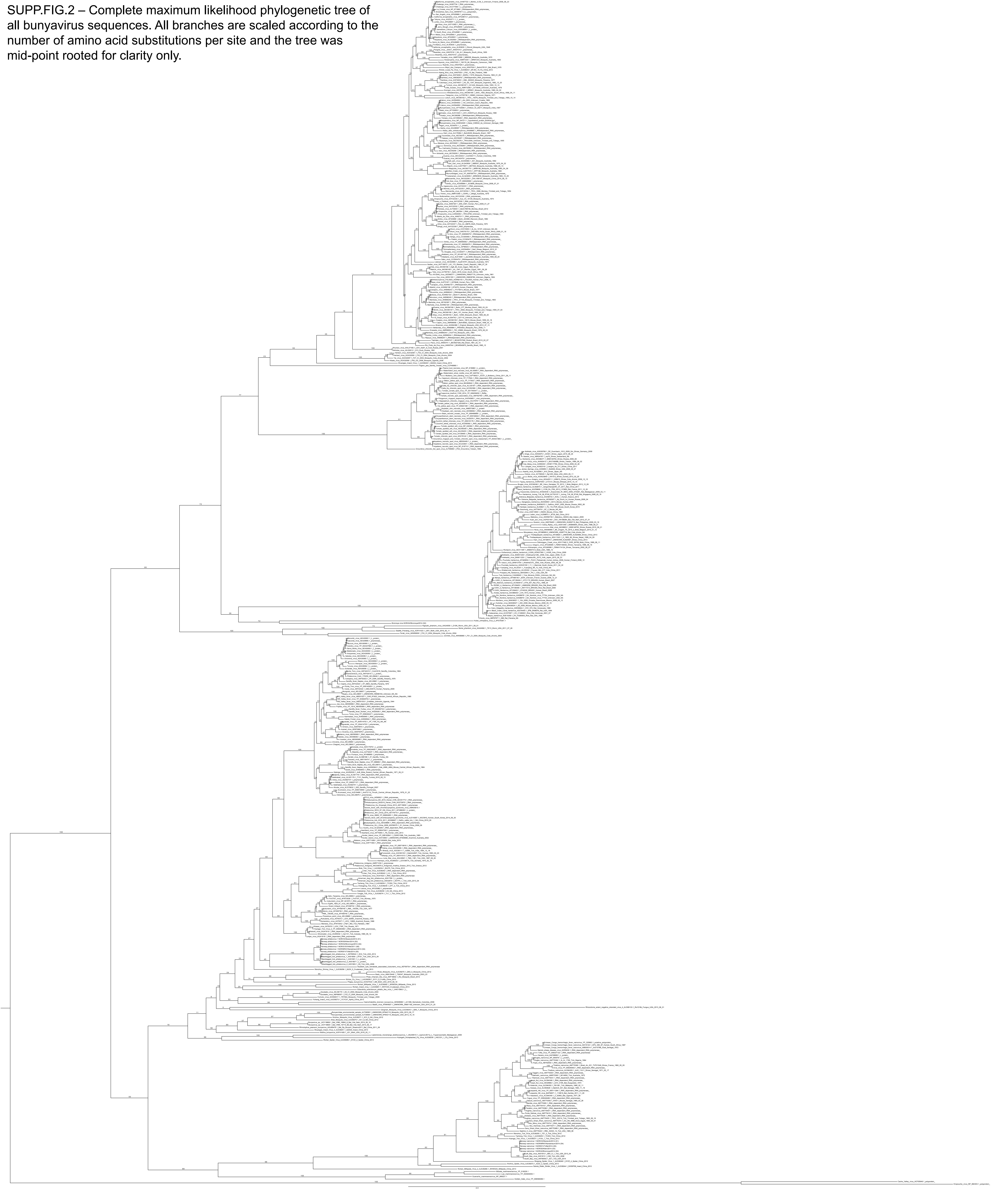

SUPPL.FIG.3 – Complete maximum likelihood phylogenetic tree of all luteovirus sequences. All branches are scaled according to the number of amino acid substitutions per site and the tree was mid-point rooted for clarity only.

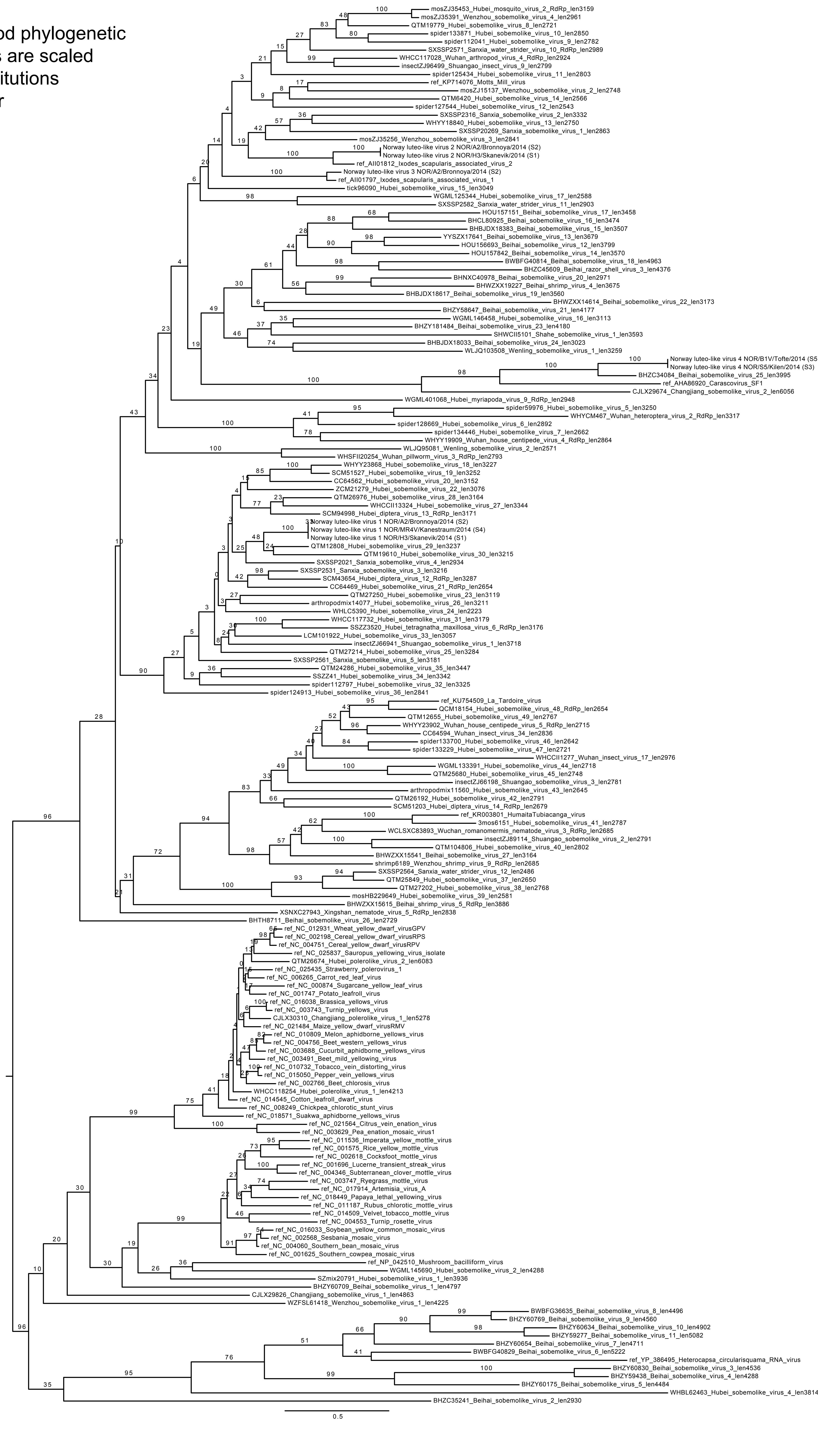

SUPPL.FIG.4 – Complete maximum likelihood phylogenetic tree of all mononegavirus sequences. All branches are scaled according to the number of amino acid substitutions per site and the tree was mid-point rooted for clarity only.

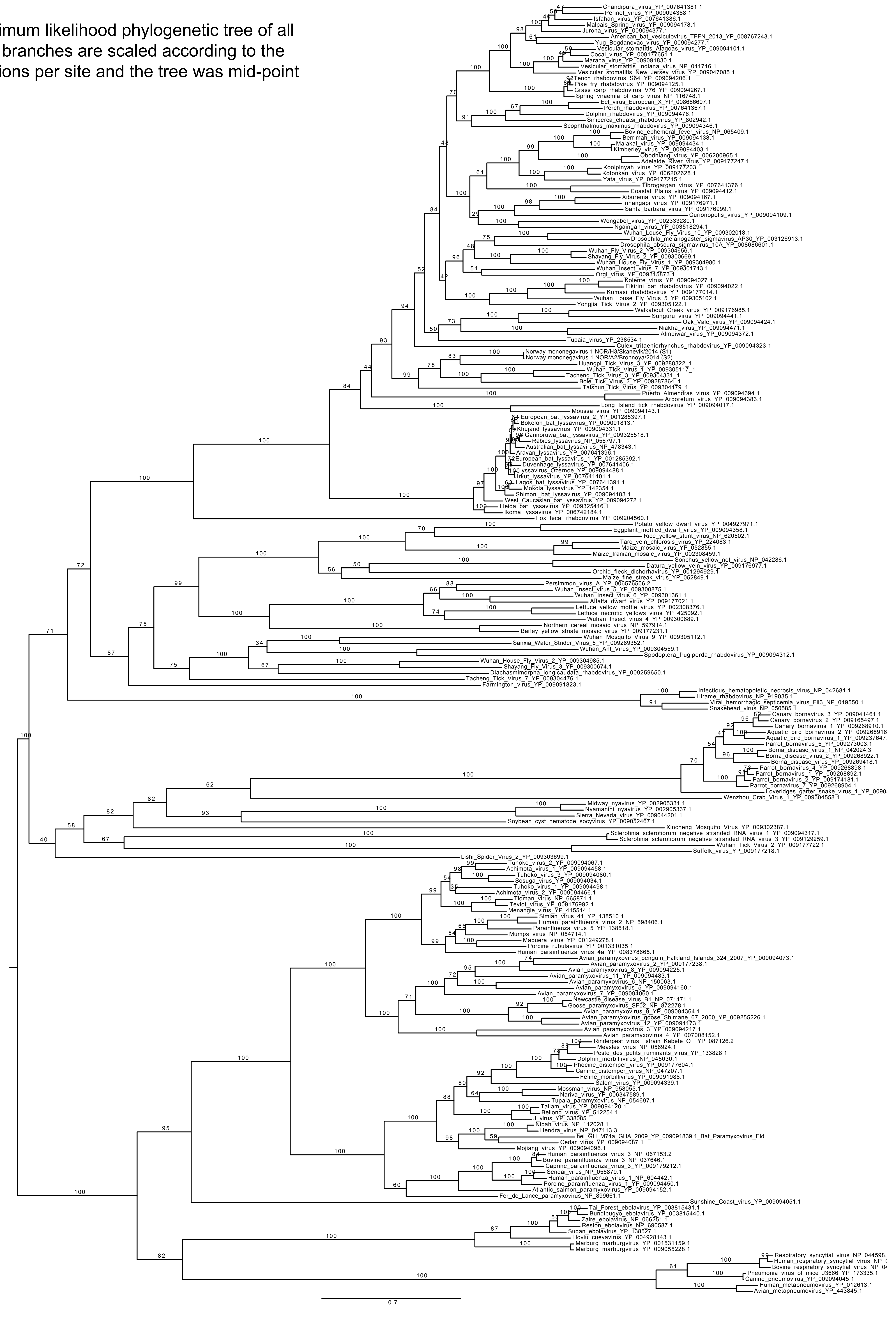

SUPPL.FIG.5 – Complete maximum likelihood phylogenetic tree of all partitivirus sequences. All branches are scaled according to the number of amino acid substitutions per site and the tree was mid-point rooted for clarity only.

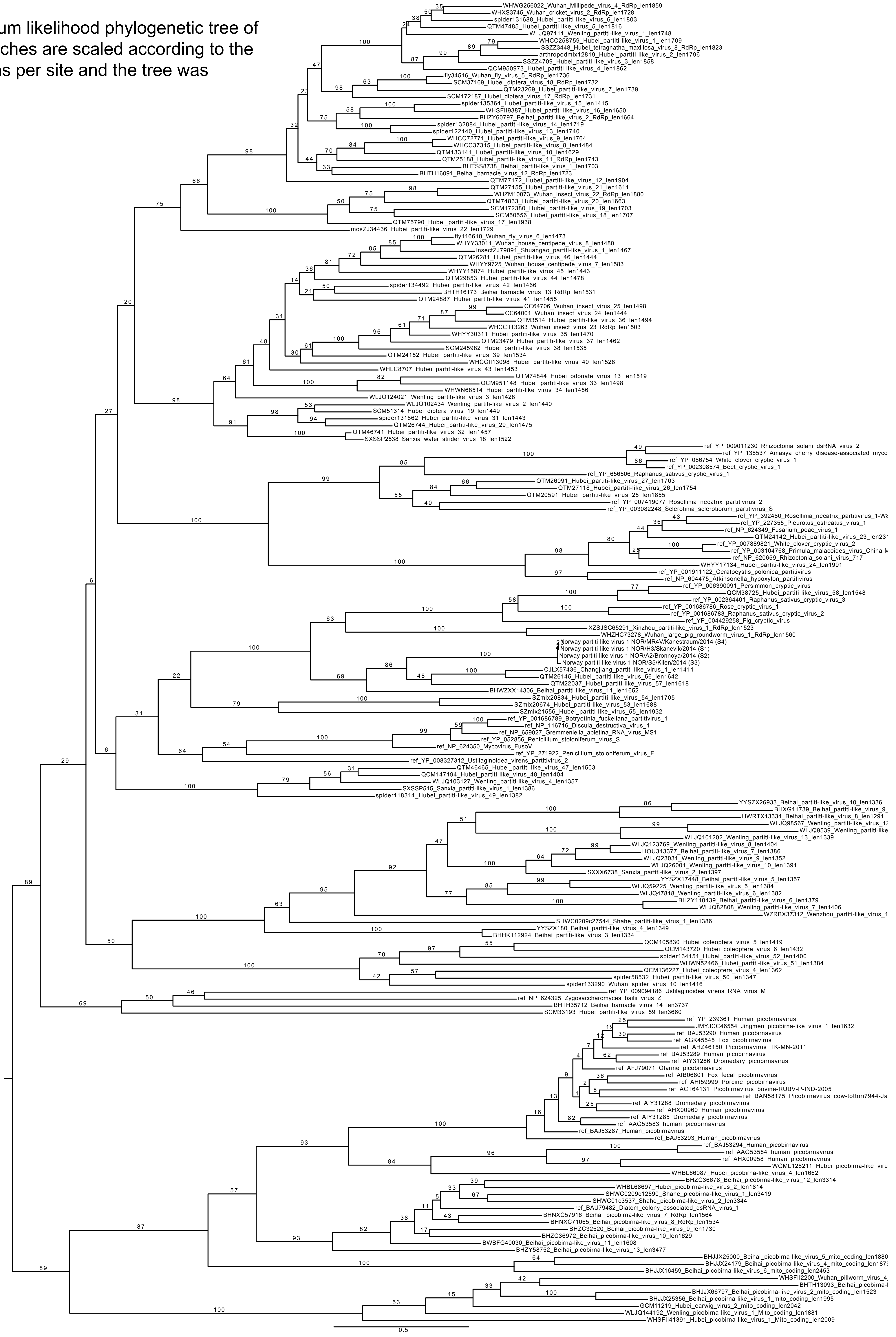

Supplementary table 1. Levels of mean sequence depth and relative frequency per virus contig per library in relation to the mitochondrial COX1 gene in *Ixodes ricinus*.

Library	Mean sequence depth[1]	Relative frequency[2]	Virus (segment) / gene
S1	635	0.03	COX1 *Ixodes ricinus*
S1	2653	0.24	Norway nairovirus 1 NOR/H3/Skanevik/2014 segment=Nucleocapsid
S1	1237	0.08	Norway phlebovirus 1 NOR/H3/Skanevik/2014 segment=Nucleocapsid
S1	955	0.34	Norway nairovirus 1 NOR/H3/Skanevik/2014 segment=L
S1	343	0.06	Norway phlebovirus 1 NOR/H3/Skanevik/2014 segment=L
S1	291	0.02	Norway luteo-like virus 1 NOR/H3/Skanevik/2014 segment=RdRp
S1	69	0.02	Norway mononegavirus 1 NOR/H3/Skanevik/2014 segment=N, P, M, G, L
S1	26	<0.00	Norway partiti-like virus 1 NOR/H3/Skanevik/2014 segment=RdRp
S1	10	<0.00	Norway luteo-like virus 2 NOR/H3/Skanevik/2014 segment=RdRp
S2	580	0.03	COX1 *Ixodes ricinus*
S2	24683	2.62	Norway luteo-like virus 1 NOR/A2/Bronnoya/2014 segment=RdRp
S2	1705	0.21	Norway nairovirus 1 NOR/A2/Bronnoya/2014 segment=Nucleocapsid
S2	878	0.07	Norway luteo-like virus 2 NOR/A2/Bronnoya/2014 segment=RdRp
S2	650	0.25	Norway nairovirus 1 NOR/A2/Bronnoya/2014 segment=L
S2	366	0.03	Norway phlebovirus 1 NOR/A2/Bronnoya/2014 segment=Nucleocapsid
S2	105	0.02	Norway phlebovirus 1 NOR/A2/Bronnoya/2014 segment=L
S2	63	<0.00	Norway luteo-like virus 3 NOR/A2/Bronnoya/2014 segment=RdRp
S2	51	0.01	Bronnoya virus NOR/A2/Bronnoya/2014 segment=L
S2	33	0.01	Norway mononegavirus 1 NOR/A2/Bronnoya/2014 segment=N, P, M, G, L
S2	20	<0.00	Bronnoya virus NOR/A2/Bronnoya/2014 segment=Glycoprotein
S2	7	<0.00	Norway partiti-like virus 1 NOR/A2/Bronnoya/2014 segment=RdRp
S3	341	0.01	COX1 *Ixodes ricinus*
S3	1957	0.09	Norway phlebovirus 1 NOR/S5/Kilen/2014 segment=Nucleocapsid
S3	1214	0.34	Norway nairovirus 1 NOR/S5/Kilen/2014 segment=L
S3	1131	0.08	Norway nairovirus 1 NOR/S5/Kilen/2014 segment=Nucleocapsid
S3	650	0.08	Norway phlebovirus 1 NOR/S5/Kilen/2014 segment=L
S3	58	<0.00	Norway partiti-like virus 1 NOR/S5/Kilen/2014 segment=RdRp
S3	10	<0.00	Norway luteo-like virus 4 NOR/S5/Kilen/2014 segment=RdRp
S4	966	0.04	COX1 *Ixodes ricinus*
S4	5993	0.36	Norway phlebovirus 1 NOR/MR4V/Kanestraum/2014 segment=Nucleocapsid
S4	3244	0.28	Norway nairovirus 1 NOR/MR4V/Kanestraum/2014 segment=Nucleocapsid
S4	1533	0.24	Norway phlebovirus 1 NOR/MR4V/Kanestraum/2014 segment=L
S4	1389	0.47	Norway nairovirus 1 NOR/MR4V/Kanestraum/2014 segment=L
S4	94	<0.00	Norway partiti-like virus 1 NOR/MR4V/Kanestraum/2014 segment=RdRp
S4	23	<0.00	Norway luteo-like virus 1 NOR/MR4V/Kanestraum/2014 segment=RdRp
S5	569	0.01	COX1 *Ixodes ricinus*
S5	8492	0.31	Norway phlebovirus 1 NOR/B1V/Tofte/2014 segment=Nucleocapsid
S5	4801	0.25	Norway nairovirus 1 NOR/B1V/Tofte/2014 segment=Nucleocapsid
S5	4732	0.45	Norway phlebovirus 1 NOR/B1V/Tofte/2014 segment=L
S5	1645	0.34	Norway nairovirus 1 NOR/B1V/Tofte/2014 segment=L
S5	45	<0.00	Norway luteo-like virus 4 NOR/B1V/Tofte/2014 segment=RdRp, Viral coat
S6	678	0.02	COX1 *Ixodes ricinus*
S6	1177	0.06	Norway phlebovirus 1 NOR/S10V/Hille/2011 segment=Nucleocapsid
S6	268	0.04	Norway phlebovirus 1 NOR/S10V/Hille/2011 segment=L

1. The number of reads per nucleotide position in relation to the total length of the contig.

2. Percentage of reads mapped to the virus contig and host reference gene, respectively.
